# The Book World of Medicine and Science

**Published:** 1894-11-03

**Authors:** 


### THE BOOK WORLD OF MEDICINE AND SCIENCE.

Doses and Strength. (London: Bailliere, Tindall, and Cox.) This little book, which is issued by the Middlesex College of Chemistry, is a table of doses and strength of all drugs mentioned in the British Pharmacopæia. It is a small book, which can easily be placed in the waistcoat pocket, and consists of about forty-two pages, printed in clear type, absolutely accurate, and most convenient not only for practitioners and medical students, but also for intending candidates of the Pharmaceutical Society's examination and diplomas. The moderate price of sixpence will deter no one who requires such friendly assistance from becoming the possessor of "Doses and Strength."

International Clinics: A Quarterly of Clinical Lectures. (Philadelphia: J. B. Lippincott Company. 1894.

There is something extremely charming in clinical lectures as a form of occasional reading. The very absence of formality in their construction differentiates them from treatises and makes them especially welcome to men engaged in practice. No introduction is necessary, no long list of authorities need be quoted; the "case" rather than the malady is the text of the lecture, just as the "case" is the problem to be solved by the practitioner. We need not, then, wonder at the popularity of this publication which quarter by quarter comes out as a goodly volume of between three and four hundred pages of clinical lectures, well printed and illustrated, bound, indexed, and ready for either reading or putting on the library shelf. The contributors hail from "the leading medical colleges of the United States, France, Great Britain, and Canada," and a very large amount of the field of medicine and surgery is covered by them. It is impossible and would be invidious to select out of so large and diverse a mass of material any individual article for special review, but we would say that while England is well represented, there are a large number of very admirable articles from teachers in the United States; and we cannot but believe that it is likely to prove a great advantage to English speaking medical men all over the world to have access to so representative a series of clinical lectures illustrating current thought and actual practice in the two great divisions of the English speaking race.

The Phonographic Quarterly Review. (London; Sir I. Pitman and Sons, 1, Amen Corner, E.C. Price 1s.)

The first number of this quarterly is a strong one, and contains several articles of interest, all of which are reproduced in shorthand, with the object of supplying stenographers with the best outlines for the expression of technical terminology. We have examined this review carefully and with interest. It is justly claimed for it that it exhibits no straining after undue brevity in the shorthand characters but every effort has been made to render the written page distinct and legible, in order that it may be read with comfort and fluency by every phonographer. A perusal of the *Phonographic Quarterly Review* must at one and the same time impart general information, promote mental culture, and increase familiarity with shorthand. The articles have been wisely confined to subjects of more or less technical character which may come within the range of professional work, and so familiarise phonographers with the best outlines, and obviate difficulties which very frequently arise at present. The Review deserves encouragement, it distinctly fills a void which has been felt for a long time, and will, we have no doubt, tend to raise the art of phonography to an even higher position than it has at present reached.

